# Fine Tuning of Globin Gene Expression by DNA Methylation

**DOI:** 10.1371/journal.pone.0000046

**Published:** 2006-12-20

**Authors:** Alon Goren, Giora Simchen, Eitan Fibach, Piroska E. Szabo, Keiji Tanimoto, Lyubomira Chakalova, Gerd P. Pfeifer, Peter J. Fraser, James D. Engel, Howard Cedar

**Affiliations:** 1 Department of Cellular Biochemistry and Human Genetics, Hebrew University Jerusalem, Israel; 2 Department of Genetics, Hebrew University Jerusalem, Israel; 3 Department of Hematology, Hebrew University Jerusalem, Israel; 4 Division of Biology, Beckman Research Institute of the City of Hope Duarte, California, United States of America; 5 Graduate School of Life and Environmental Sciences, University of Tsukuba Tsukuba, Japan; 6 Laboratory of Chromatin and Gene Expression, The Babraham Institute Cambridge, United Kingdom; 7 Department of Cell and Developmental Biology, University of Michigan Medical School, Ann Arbor Michigan, United States of America; University of Munich, Germany

## Abstract

Expression patterns in the globin gene cluster are subject to developmental regulation in vivo. While the γ^A^ and γ^G^ genes are expressed in fetal liver, both are silenced in adult erythrocytes. In order to decipher the role of DNA methylation in this process, we generated a YAC transgenic mouse system that allowed us to control γ^A^ methylation during development. DNA methylation causes a 20-fold repression of γ^A^ both in non-erythroid and adult erythroid cells. In erythroid cells this modification works as a dominant mechanism to repress γ gene expression, probably through changes in histone acetylation that prevent the binding of erythroid transcription factors to the promoter. These studies demonstrate that DNA methylation serves as an elegant in vivo fine-tuning device for selecting appropriate genes in the globin locus. In addition, our findings provide a mechanism for understanding the high levels of γ-globin transcription seen in patients with Hereditary Persistence of Fetal Hemoglobin, and help explain why 5azaC and butyrate compounds stimulate γ-globin expression in patients with β-hemoglobinopathies.

## Introduction

Genome wide DNA methylation is set up during early development and then maintained in a semi-conservative manner through every cell division [1]. The presence of methyl groups apparently serves as a molecular signal that can direct the packaging of DNA into a closed inaccessible chromatin conformation [2] and bring about the local deacetylation of histones H3 and H4 [3]. Because of these observations, it has been postulated that methylation may serve as a global mechanism for repression of basal transcription [4]. In keeping with this organization, many tissue specific genes are found methylated in almost all cell types of the organism, but undergo developmentally regulated demethylation in their cell type of expression [5]. Little is known about the role of DNA methylation in this differentiation process.

A good system for studying gene specific methylation is the human β-globin locus on chromosome 11 which is composed of five different β-type genes that are expressed in a regulated manner during development [6]. In non-erythroid cells, the entire locus replicates late in S phase [7]–[9] and is packaged into a closed DNaseI-insensitive chromatin conformation, where all of the individual genes are methylated. In contrast, during erythroid cell specific differentiation, the full locus undergoes an “opening” process, becoming early replicating and generally DNaseI sensitive [10], but only specific genes undergo demethylation and actually become transcriptionally active. Thus, for example, γ-globin genes are preferentially active in the fetal liver, while mainly the β gene is active in adult erythroid cells where both γ^A^ and γ^G^ remain methylated [11] and silenced in a process that appears to be mediated by MBD2 [12].

Using a genetic approach in transgenic mice we demonstrate that DNA methylation plays a local role in preventing the activation of γ globin in adult erythroid cells, and this effect is mediated by histone deacetylation and the prevention of factor binding. These findings provide a strong experimental basis for approaches aimed at inducing γ expression in cases of β-Thalassemia through treatments that bring about DNA demethylation or histone acetylation. Our results also suggest that demethylation may be an obligatory step in the molecular mechanisms that bring about the abnormal expression of γ globin seen in Hereditary Persistence of Fetal Hemoglobin.

## Results

### Experimental strategy

**Figure 1:**
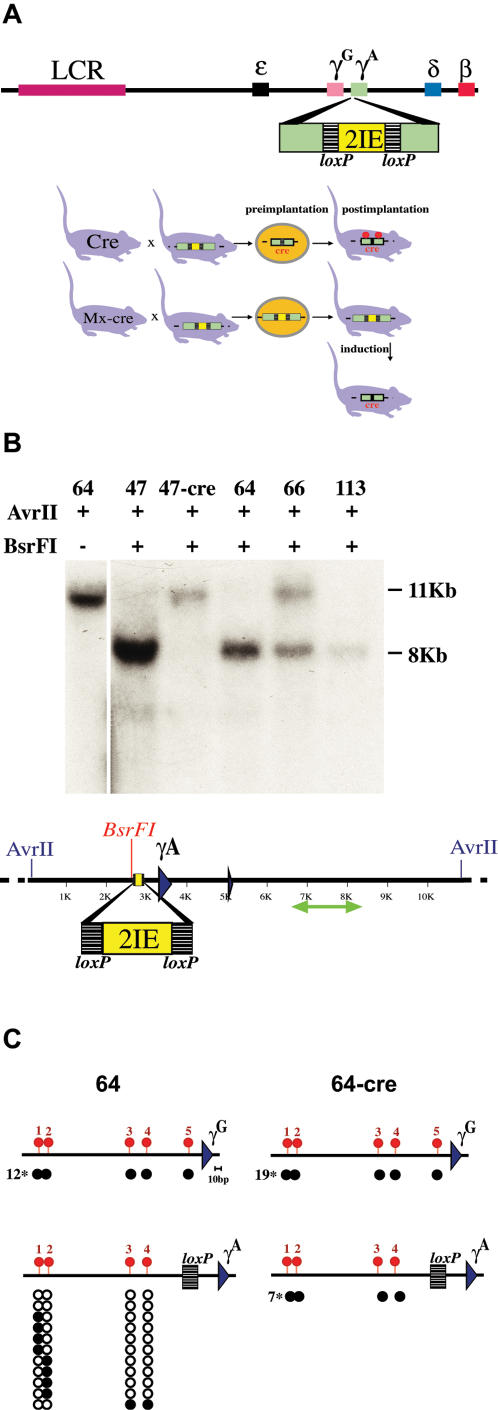
Programmed methylation of the human γ^A^-globin promoter **A.** Two IEs (yellow) bounded by loxP elements (stippled black) were inserted into the γ^A^ promoter region of a YAC containing the human β-globin locus [18], and this was used to generate transgenic mice that were then crossed with two different cre-expressing lines. In the first line (Cre) [19], cre is expressed (red) prior to implantation. In mice carrying this construct the IE is deleted before the wave of de novo methylation, and surrounding CpG sites thus become methylated (red circles). The second cre-expressing line carries interferon-inducible cre [20]. In these mice (Mx-cre), the IE remains present during implantation and protects adjacent regions from methylation. By treating adult animals with polyI-polyC, cre activity could be induced (red) and the IE removed, generating an unmethylated version of the transgene. **B.** Tail DNA from four transgenic founders (lines 47, 64, 66 and 113) was cut with AvrII with or without the methylation sensitive restriction enzyme BsrFI, and subjected to Southern blotting using a radioactive probe (green arrow). Line 47 was crossed with cre mice and offspring analyzed in the same way. **C.** The region upstream of the γ^A^ and γ^G^ coding sequences (blue triangles) was analyzed for CpG methylation using the bisulfite technique. To this end we generated MEFs from line 64 (IE present at time of implantation) and infected them with an adenovirus that expresses Cre activity. Alternatively, we derived MEFs from line 64 mice crossed with Cre (IE removed prior to implantation). Five CpG residues were analyzed for methylation in the γ^G^ promoter. The normal γ^A^ promoter also carries 5 CpGs, but one is replaced by the inserted loxP element. Following bisulfite treatment, PCR products were cloned and subjected to sequencing. Each row represents a single molecule. Black circles indicate a methylated CpG, whereas open circles indicate a lack of methylation. The numbers in the margin indicate how many clones were fully methylated.

In order to determine the precise role of DNA methylation in the repression of γ-globin gene expression, we devised a biological approach for controlling local methylation. For this purpose we used a (Yeast Artificial Chromosome) YAC transgene carrying the entire human β-globin locus that has been shown to be properly regulated in the mouse [13], [14]. During early development, at the time of implantation, CpG island regions are recognized and protected from the process of de novo methylation [15]. This is accomplished through the involvement of cis acting sequences [16], [17], and one such element (IE—Island Element) identified in the Aprt locus, has been shown to work dominantly to prevent methylation of transgenes in vivo [3]. Preliminary experiments indicated that the insertion of two tandem copies of the IE near the γ-globin promoter could generate a relatively large (1 kb) unmethylated region that was then maintained in all somatic cells of the mouse (see Materials and Methods). On the basis of this observation we engineered a duplicated IE sequence flanked with LoxP sites 40 bp upstream to the γ^A^ -globin gene transcription start site in a 150 kb YAC which carries the entire human β-globin locus [18], and this construct was used to generate transgenic mice (Figure 1A). Four founder animals with intact YACs were obtained, and Southern blot analysis showed that 3 of them (47, 64, 113) were completely unmethylated at one selective restriction site in the γ^A^ gene promoter, indicating that this strategy was successful (Figure 1B). In one transgenic animal (66), the promoter remained partially methylated.

In order to manipulate DNA methylation and thereby test its effect on γ^A^ transcription, transgenic mice were crossed with two types of cre-expressing animals. The first line of cre mice (Cre) contain an expression vector which is constitutively active in all cell types from very early stages in development [19]. In F1 animals generated from this cross, the IE would be removed prior to implantation and, as a result, the γ^A^ gene becomes methylated (see Figure 1). The Mx-Cre mouse, in contrast, contains an interferon-inducible cre gene [20]. In F1 animals from this cross, the γ^A^ gene should be protected from de novo methylation at the time of implantation. In the adult, the IE can be removed by treatment with interferon, but the gene should still remain unmodified. Thus, using this strategy, it should be possible to generate matched transgenic mice with a fixed integration site whose only difference is the DNA methylation state at the γ^A^ promoter throughout the organism [21].

### Effect of DNA methylation on gene expression

Using the globin YAC transgene, it was now possible to determine the effect of DNA methylation on transcription of the γ^A^ gene in both non-erythroid and erythroid cells. As non-erythroid cells, we used mouse embryonal fibroblasts (MEFs) generated from Cre or Mx-Cre transgenic (line 64) crosses. The bisulfite sequencing technique was used to measure the methylation state at the γ^A^ and γ^G^ promoters. In cells in which the IE was removed pre-implantation (Cre), the γ^A^ gene was found to be completely methylated at 4 upstream CpG sites. In contrast, cells that retained the IE during implantation (Mx-Cre) were highly unmethylated in this same region (Figure 1C). The γ^G^ promoter provided an important control that remained methylated in all MEFs from transgenic mice.

**Figure 2:**
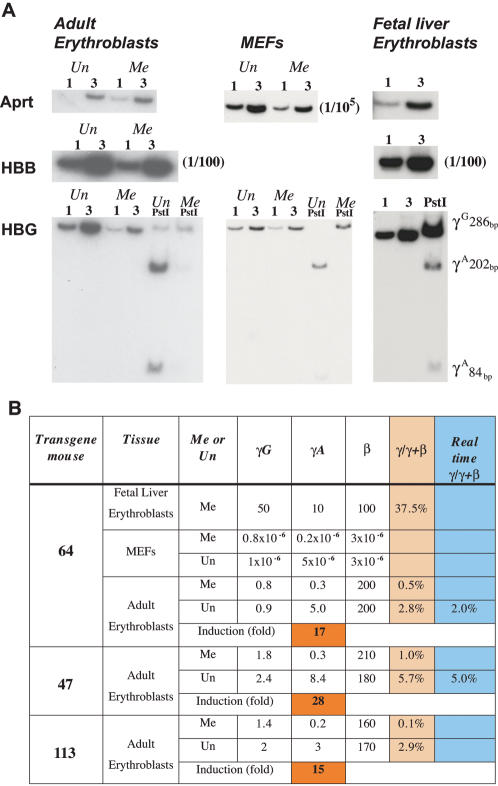
Effect of DNA methylation on gene expression A. RNA from purified adult or fetal liver erythroblasts and MEFs from Cre (γ^A^ promoter methylated) or Mx-cre (γ^A^ promoter unmethylated) crossed transgenic (lines 47, 64 or 113)) mice were subjected to semi-quantitative RT-PCR on 1 or 3 µl samples to detect human β (HBB) or γ(HBG)-globin RNA. We measured Aprt expression to control for the amount of cDNA in the reaction. Genes expressed at high levels were quantitated by diluting the input sample (e.g. 1/10^5^ for Aprt in MEFs). In order to distinguish between the two γ genes, we took advantage of a PstI restriction site that is present in γ^A^ and not γ^G^ [51]. A smaller induction of γ^A^ was observed in line 66 which is only partially unmethylated at the promoter (data not shown). The degree of IE excision in purified erythroblasts from induced Mx-cre carrying mice was found to be >90% by PCR analysis. The data for fetal erythroblasts was taken from an animal with a “methylated” γ^A^ gene, but we have demonstrated that it is indeed unmethylated in fetal liver (data not shown), as expected. B. Quantitative analysis of RNA. Levels of globin expression were obtained from dilution analysis and compared to Aprt (set to 1) in the same cells. Each experiment was repeated 2–3 times (coefficient of variance = 14%). γ/γ+β was calculated on the basis of total γ globin (γ^A^+γ^G^). Results (average of 3 experiments) for real time PCR are included for some samples. The degree of γ globin induction is shown for adult erythroblast cells.

Semi-quantitative RT-PCR was used to determine the level of expression of the γ as well as β-globin genes. We took advantage of a unique restriction enzyme site within the γ^A^ gene to distinguish between γ^A^ and γ^G^ transcripts. We found that the unmethylated γ^A^ gene is transcribed at much higher levels (about 20 fold) than the methylated copy, while both the γ^G^ and β-globin genes are expressed at similar levels (Figure 2). These results are consistent with previous studies showing that DNA methylation of γ gene promoters inhibits transcription in transfected fibroblasts [22]. It should be noted that these steady state levels of RNA in MEFs are about 8–9 orders of magnitude less than that in fetal liver or adult erythroblasts. Most of this inhibition is clearly independent of DNA methylation.

In order to examine the role of DNA methylation in the control of γ-globin transcription in erythroid cells, we isolated adult spleens from transgenic animals (lines 47, 64, 113) and separated out erythroblasts using a cell-type specific antibody affinity column. In these cells, the β-globin gene is expressed at very high levels (Figure 2B). While the γ-globin sequences are transcribed at a low level compared to β-globin, this is still about 6 orders of magnitude higher than that measured in non erythroid cells. This increased expression is presumably due to the fact that this locus is in a more open overall chromatin conformation in these cells. It should be noted, however, that this amount of γ^A^-globin RNA is still considerably lower than the levels produced in fetal liver. Undermethylation of the γ^A^ gene promoter in spleen erythroblasts brings about a striking 20-fold average increase in the activity of this gene as compared to its methylated counterpart in three independent transgenic founders. In contrast, the constitutively methylated γ^G^ promoter remained repressed, and this serves as an internal negative control for the induction seen with γ^A^.

Although the normal percentage of γ globin expression in human erythroblasts is about 5–10% of the total (γ+β) (http://www.genecards.org/index.shtml; http://symatlas.gnf.org/SymAtlas), this ratio appears much lower (1%) in our mice, and this is, in fact, consistent with previous data from other transgenic animals carrying similar constructs (0.3–3%) [14], [23]. In the absence of DNA methylation on the γ^A^ promoter, γ globin expression ratio increases (to a level of 3–6%), but if we assume that the γ^G^ gene is affected by demethylation to the same extent, total γ/γ+β would actually be expected to reach levels of 10–25%, which is approximately the same order of magnitude as that seen in fetal liver (Figure 2 and or cases of transgenic HPFH (30–65%)where both the γ and β genes are active [24]. It thus appears that DNA methylation is serving as a local mechanism to repress the activity of γ-globin genes in adult erythroid cells.

### Effect of DNA methylation on histone modification

One of the mechanisms by which DNA methylation inhibits gene expression is by bringing about the deacetylation of local histones [3]. We therefore examined the histone modification pattern of nucleosomes positioned in the promoter region of the γ^A^ and γ^G^ genes in vivo. This was carried out by isolating mononucleosomes from fibroblast or erythroblast cells and subjecting them to Chromatin Immunoprecipitation (ChIP) analysis using antibodies against Ac-H4. The active mouse β-actin and inactive α actin genes were used as matched controls for verifying the efficacy of immunoprecipitation.

**Figure 3:**
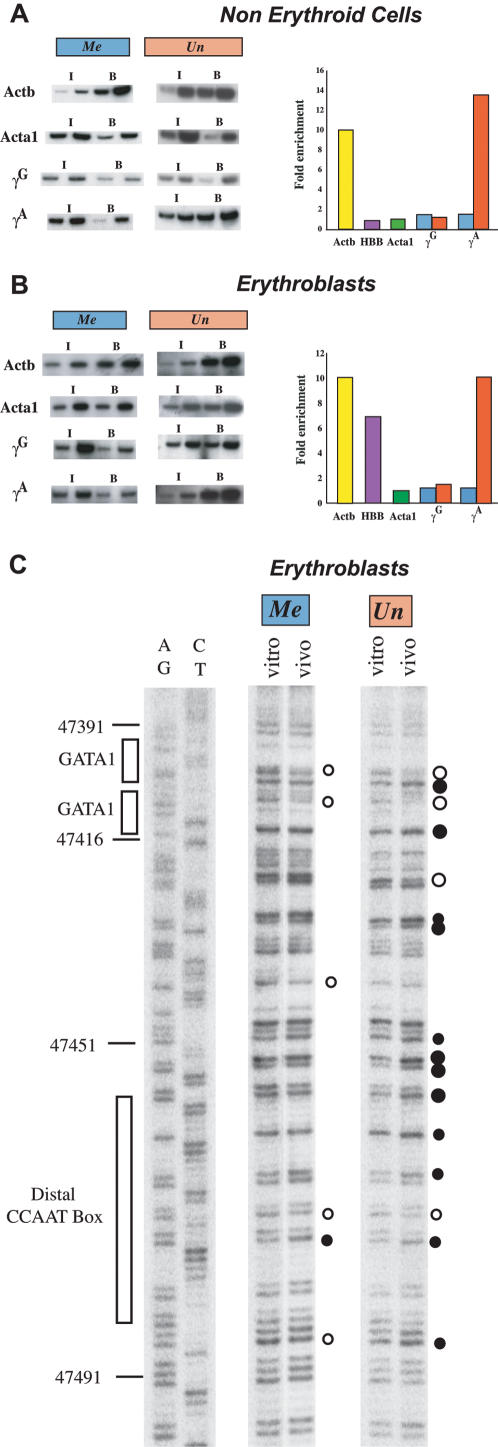
Effect of DNA methylation on promoter structure Mononucleosomes were prepared from either non-erythroid (**A**) or erythroid (**B**) cells taken from Cre (methylated) and Mx-cre (unmethylated) founder (64) transgene crosses and subjected to ChIP analysis using anti-Ac-H4. Input (I) and bound (B) DNA fractions were used for semi-quantitative PCR on 1 or 3 µl samples using primer sets specific for the γ^G^ or loxP-inserted γ^A^ promoter regions. For each ChIP preparation, the Acta1 gene (green) was assayed as a negative control and the Actb gene (yellow) as a positive control. The results are summarized in graphic form after normalizing to Acta1 (green) enrichment (set at 1.0) (coefficient of variance = 17%). The results for the Cre (blue) and Mx-cre (red) mice are shown. Results for β globin are presented for comparison. The data shown for non-erythroid cells was obtained using mononucleosomes from MEFs, but the graph summarizes results from 3–4 ChIP experiments on both MEFs and lymphocytes. (**C**) In vivo footprinting of γ promoter. Erythroblasts (in vivo) or purified erythroblast DNA (in vitro) from mice carrying either a methylated or unmethylated γ^A^ promoter were treated with DMS. LMPCR gel. Maxam-Gilbert lanes (AG and CT) are sequencing controls. The numbers are according to PubMed accession number NG_000007. Putative protein factor binding regions (rectangles) [26], DMS footprints, as in vivo protected (open circle) or hyper-reactive (closed circle) nucleotides, are indicated. The sizes of the circles represent relative differences in footprint intensities. DMS footprinting on spleen lymphocytes did not show any hyperreactive sites on unmethylated DNA, but did reveal some slight reactivity over the distal CCAAT box on methylated DNA.

As expected, the methylated γ^A^ gene promoter in non-erythroid cells is packaged in nucleosomes containing deacetylated histone H4, in a manner similar to other repressed genes. Strikingly, in the absence of DNA methylation, this region becomes acetylated, reaching the same maximum levels probably as a result of methyl binding proteins [12] associated with transcriptionally active genes such as β-actin or β-globin in erythroid cells (Figure 3A). Identical findings were observed for methylation of H3(K4) (data not shown), which is also known to be correlated with gene activity. This change in the histone modification pattern is surprising in light of the fact that γ-globin is expressed at such low levels in non-erythroid cells, suggesting that as opposed to other repression mechanisms, it is DNA methylation that plays a dominant role in setting up or maintaining histone acetylation at this locus.

Histone acetylation was also examined in adult erythroblasts purified from spleen. In these cells, the γ genes are expressed at levels 6 orders of magnitude greater than that seen in fibroblasts. Despite this, both the γ^G^ and γ^A^ promoters are packaged with nucleosomes containing deacetylated histone H4 (Figure 3B). This is most likely due to the presence of DNA methylation, since removal of these methyl moieties brings about a dramatic increase in both the acetylation of H4 and methylation of H3(K4) (Figure 3B and data not shown). This suggests that in erythroid cells, it is basically DNA methylation which keeps the γ genes packaged in their deacetylated form, and this probably accounts for their suboptimal level of expression.

### Effect of DNA methylation on protein binding

One of the major effectors of globin expression in erythroid cells is the presence of highly specific transcription factors such as GATA1, CP1/NF-Y and NFE3 that recognize defined sequence motifs present at many sites within the globin locus, including the γ^A^ promoter [25]–[28]. In order to test whether DNA methylation plays a role in these interactions, we carried out in vivo footprint analysis in both adult erythroid cells and lymphocytes derived from the spleen. As expected, no significant footprints were observed in the lymphoid cells (data not shown). Similarly, footprints were also absent in normal adult erythroid cells in which the γ^A^ promoter is methylated. In contrast, when these methyl groups are absent, strong footprints over both the GATA-1 and distal CP1/NF-Y (CCAAT box) binding sites were observed (Figure 3C). It should be noted that these elements themselves do not contain any CpG sites, so it is unlikely that binding is inhibited directly by methyl groups. Rather, it appears that these specific protein-DNA interactions are prevented at the nucleosome level by local methylation-mediated histone deacetylation.

### DNA methylation in HPFH

**Figure 4:**
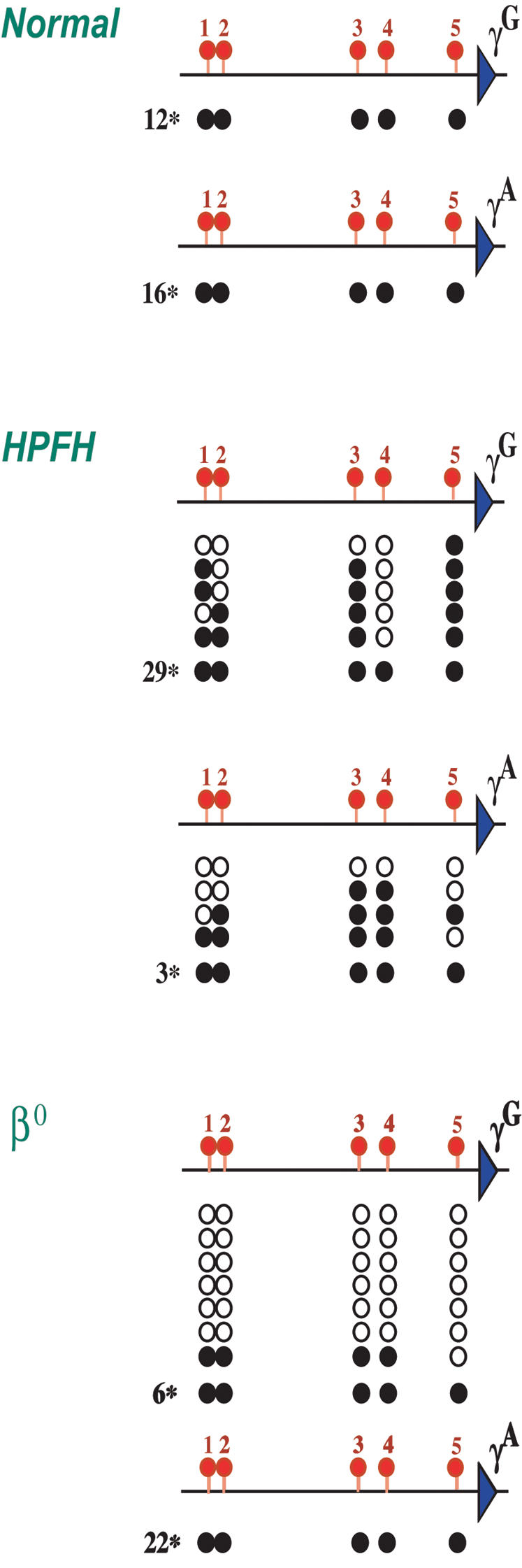
γ promoter methylation pattern in patients with HPFH The γ^A^ and γ^G^ promoter regions were analyzed for CpG methylation on individual clones by the bisulfite technique. To this end we used Fibach culture erythroblasts derived from normal individuals, as well as heterozygote HPFH1 or β^0^ Thalassemia (IVS-II-I, G⇑A) patients. Black circles indicate methylated CpGs, whereas open circles indicate a lack of methylation. The numbers in the margin indicate how many clones were fully methylated.

In patients with Hereditary Persistence of Fetal Hemoglobin (HPFH) the γ genes are expressed at unusually high levels in adult erythroid cells [29]. Our results on the role of DNA methylation at this locus strongly suggest that whatever the molecular mechanism at the source of this disease, activation of the γ-globin genes should only be possible if the promoter is demethylated. In order to test this hypothesis, we prepared erythroblast cultures [30] from HPFH patients with different genetic lesions, and then isolated DNA for bisulfite analysis of the γ^A^ and γ^G^ genes. In normal pro-erythroblasts, all 5 CpG sites in the γ promoter region are fully methylated. In contrast, we observed under-methylation in both the γ^A^ (4/7) and γ^G^ (5/34) promoters in a patient heterozygous for HPFH1 [31], [32] (Figure 4). This skewed pattern is in keeping with measurements showing that these patients also have a higher than normal ratio of γ^A^ to γ^G^ expression [32].

Similar bisulfite results were obtained for pro-erythroblast DNA from a patient with β° Thalassemia (IVS-II-I, G⇑A) [32] who expresses high levels of γ^G^ -globin, and in this case we detected massive under-methylation almost exclusively at the γ^G^ promoter (Figure 4). These findings are consistent with earlier experiments showing under-methylation at a single restriction site in the γ promoter regions from HPFH patients [33], [34]. Taken together, these results strongly suggest that whatever the mechanism [35], [36] involved in enhancing γ gene transcription, each case of HPFH is probably associated with molecular changes that can bring about local demethylation or perhaps histone acetylation in adult erythroid cells. In further support of this, we find that DNA methylation actually affects protein binding specifically at the exact same sites in the γ promoter that are frequently involved in HPFH [28].

## Discussion

In non-erythroid cells, the entire globin locus is associated with heterochromatin [37], [38] and packaged in a closed chromatin conformation characterized by a lack of sensitivity to DNaseI [10] and late replication timing [8]. In addition, the γ promoters carry methyl groups that were initially put on at the time of implantation and then maintained through subsequent cell divisions. These mechanisms apparently serve to make the locus compact and highly inaccessible to nuclear proteins such as RNA polymerase and its accompanying basal transcription factors (Figure 2B). For this reason, synthesis from the γ promoters initiates very infrequently. By genetically preventing normal developmentally-regulated DNA methylation at this site, we were able to selectively remove a single layer of protection, thus allowing the transcription machinery to increase initiation of RNA synthesis at the promoter by about 20 fold. We have previously demonstrated that this is accomplished through methyl binding proteins that locally recruit histone deacetylase [3]. Our results strongly suggest that in the absence of methylation, the auxiliary repression layers, which evidently operate through very different mechanisms, are not able to bring about local histone deacetylation.

Previous studies had shown that the human globin locus is programmed to be turned on regionally in erythroid cells generated at different stages of development. This is mediated by an increase in chromatin accessibility (DNaseI sensitivity) over the entire locus [10], as well as a shift to early replication timing [39], an epigenetic change that also influences chromatin structure [40]. As part of the normal process of adult erythroid differentiation, the β-globin gene undergoes local demethylation [11], and this probably represents the last stage necessary to allow the binding of erythroid trans acting transcription factors.

Although the γ-globin genes do not undergo demethylation in adult erythroid cells and are still packaged with deacetylated histones, chromatin changes in cis make these genes about 10^6^ fold more likely to be transcribed, but this is still not sufficient to allow the synthesis of γ-globin at maximum levels. By manipulating the locus so that the γ^A^ gene is unmethylated, however, the last repression barrier is removed and this gene can then interact with the strong transcription factors already present in erythroid cells, thereby allowing near maximal levels of RNA synthesis. Interestingly, these factors fail to react with similar β globin promoter elements in fetal erythroid cells [26], strongly suggesting that this may also be due to DNA methylation. Thus, in addition to being a basal repression mechanism in non-erythroid cells, DNA methylation also works as a selective regulator in erythroid cells. Since DNA methylation plays a similar role in the immune system [41], it is possible that this represents a general fine-tuning mechanism for controlling expression within gene clusters.

Several studies have shown that the symptoms in β-Thalassemia can be partly alleviated by treatment with DNA demethylation [42] or histone acetylation agents [43], presumably by bringing about increased synthesis of γ-globin. Our results provide molecular evidence that DNA methylation is indeed the major barrier to γ expression in adult erythroid cells, and provide the impetus to develop more specific drugs for removing methylation from the γ promoters.

## Materials and methods

### Cloning

**Figure 5:**
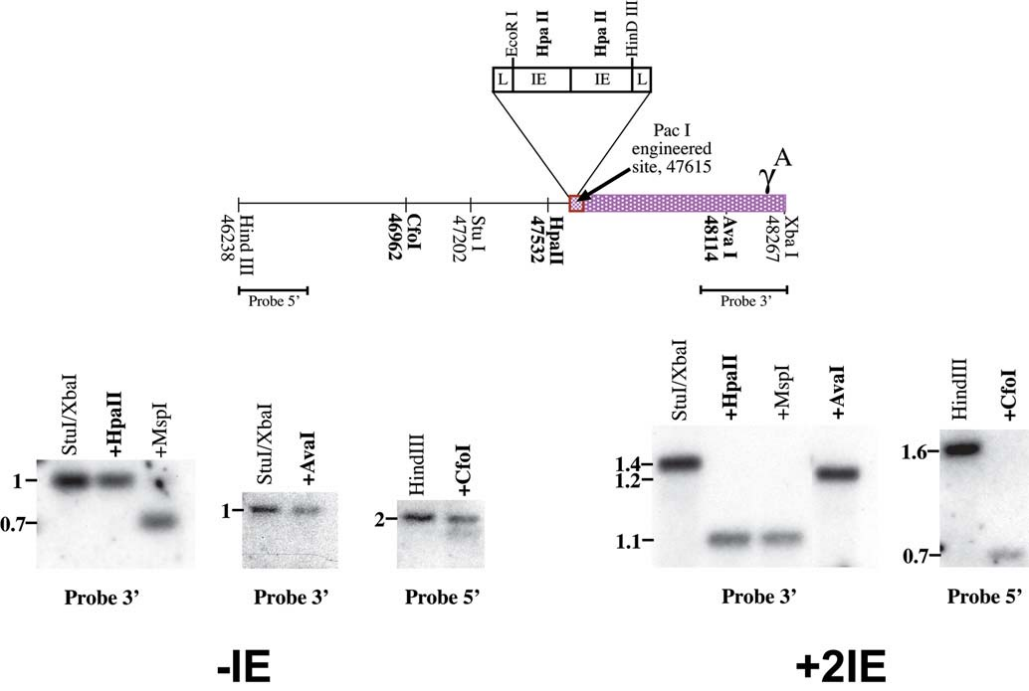
IE induces undermethylation in transgenic mice We made transgenic embryos (12.5 dpc) using either a plasmid that contains the normal promoter of the human γ^A^-globin gene (-IE), or a plasmid that contains the promoter of the human γ^A^-globin gene modified to harbor a dimer of the IE bounded by two lox elements (+2IE) 40 bp upstream to the γ^A^ transcription start site, and tested total founder embryonic DNA for methylation at specific restriction sites by southern blot analysis. This plasmid (+2IE) was later used as a template for homologous recombination in the YAC (see methods). The map indicates the restriction site locations according to PubMed accession number NG_00007. Bold marked restriction enzymes are methylation sensitive. Total founder embryo DNA was digested with HindIII alone or together with CfoI, or alternatively with StuI/XbaI alone or together with HpaII, MspI or AvaI. Southern-blot analysis was performed using probes 5′ or 3′ as labeled. The sizes of expected bands are indicated on the autoradiogram. Data shown are representative of results obtained with several transgenic founder mice for each construct. From this experiment, we conclude that a double IE is capable of inducing undermethylation over a distance of at least 500 bp in each direction.

The 120 bp IE from the hamster Aprt gene [21] was inserted as a dimer bounded by two lox elements into an engineered PacI restriction site 40 bp upstream to the human γ^A^-globin gene transcription start site (Figure 5). The HindIII/XhaI fragment was then cloned into the yeast integrative plasmid pRS306 that was then inserted by homologous recombination into the human β-globin YAC-harboring yeast cells A201F4.3 [18]. Colonies were subsequently screened for growth on plates lacking uracil. Correct integration was verified by Southern blot analysis. Plasmid sequences were removed by induction of “looping out” through growth on 5FOA and correct excision was verified by Southern blot analysis.

### Transgenic mice and methylation analysis

YAC DNA was purified from pulse-field gels according to standard protocols [44] and injected into fertilized mouse eggs that were transferred to foster mothers. Littermates were screened for the β-globin YAC transgene by PCR, and positive samples verified by Southern blot. All three transgenes (lines 47, 64, 113) used for analysis were found to be completely intact and present as a single copy. Founders were crossed with Cre [19] or Mx-Cre mice [20]. To remove the IE sequences after birth Mx-Cre X β-globin YAC F1 mice were injected 4 times with polyI-polyC (Sigma) on alternate days. MEFs (passage number 3–4) carrying a methylated γ^A^ gene were prepared from Cre embryos. MEFs carrying an unmethylated γ^A^ gene were prepared from Mx Cre mice and then transfected with a type 5-cytomegalovirus Cre vector [45] to remove the IE element. Methylation patterns in non erythroid cells were analyzed either by Southern blot or bisulfite methylation analysis as described [46]. Following treatment, the DNA was amplified using the PCR primers forward -1 (5′-TTGTTTGAAAGGGTTTTTGGTTAAATTTTATTT-3′) and reverse-1 (5′-TCCTAATCACCAAAACCTACCTTCCCAAAA-3′) and subsequently with the nested primers forward -2 (5′-TTTTGGTTAAATTTTATTTATGGGTTGGTTAGTT-3′) and reverse-2 (5′-AACTTATAATAATAACCTTATCCTCCTCTATAAAATA-3′). The resulting products were cloned using the pGEM-T Easy Vector System I kit (Promega) and individual colonies then sequenced. Methylation patterns in purified erythroid cells were analyzed by digestion with methylation sensitive restriction enzyme followed by amplification by PCR.

### Semiquantitative RT-PCR

Erythroblasts from fetal liver and adult spleen of Cre or Mx-cre crossed transgenic mice were isolated by MACS (Magnetic Activated Cell Sorter) with biotinilated α-ter119 antibodies (PharMingen). Mouse embryonic fibroblasts (MEFs) were derived from Cre or Mx-cre crossed 12.5 dpc transgenic embryos. RNA was extracted with TriPure Isolation reagent (Roche), treated (250 ng) with DNaseI (Promega) and converted to cDNA using the Molony murine leukemia virus reverse transcriptase (Promega) and random hexanucleotide pd(N)_6_ primers (Pharmacia) in a reaction volume of 50 µl under conditions recommended by the manufacturer. 1 or 3 µl of the cDNA was used for PCR reactions (95°C for 1 min, 58°C for 1 min, 72°C for 1 min) in the presence of ^32^PαdCTP (Amersham) using appropriate primer pairs [47]. These primers were designed to span intron-exon junctions in order to distinguish between cDNA and genomic DNA. RT-PCR fragments were separated on 5% or 7% polyacrylamide gels and exposed for autoradiography. The relative amount of γ^A^ and γ^G^ cDNA was defined by digestion with the restriction enzyme PstI that cuts only the γ^A^ PCR product. The level of γ and β-globin transcription was measured by phosphor-imager analysis of diluted samples after normalizing for Aprt.

### Chromatin Immunoprecipitation (ChIP)

Nuclei were prepared from cell culture or purified mouse spleen erythroblasts, treated with an HDAC inhibitor (PMSF) and digested by MNase (micrococcal nuclease) to generate mono-nucleosomes that were separated on a sucrose gradient [48]. Immunoprecipitation (IP) was carried out using anti-acetylated histone H4 or anti me-H3(Lys4) antibodies (Upstate Biotechnologies), and the bound fraction purified by protein A-Sepharose chromatography (Sigma). Input and Bound DNA fractions were analyzed by semiquantitative PCR using two concentrations. Primer sequences are available upon request.

### In vivo footprinting

In vivo Dimethyl Sulfate (DMS) footprinting and ligation-mediated PCR (LMPCR) was performed as described earlier [49], [50], except that the in vivo DMS treatment was done on erythroid cells in cell suspension. In a 50 ml conical tube, 10–25 million cells were suspended in 10 ml of culture medium without bovine serum. DMS was added to a final concentration of 0.2%, and the cells were incubated at room temperature for 5 min. 40 ml of ice-cold PBS was added to stop the DMS reaction and the cells were centrifuged quickly (250 g, 3 min), washed two times with 30 ml of ice-cold PBS before DNA preparation. For the LMPCR experiment we designed the first primers in such way that they distinguished between the γ^G^ and lox-P inserted γ^A^ promoters. Primer extension was then carried out with either L1n: 5′-GCGTCTGGACTAGGAGCTTTTAAT-3′ or L1: 5′-AAGTAACGCATTTGCT GGAAG-3′ (which includes the IE element). Primer, L2: 5′-TTAATCTCAGACGTTCCAGAAGCGAGTGTG-3′ and LP25 linker primer were used for amplification. The run off hybridization probe was made on PCR fragment L3: 5′-CAGAAGCGAGTGTGTGGAACTGCTGAA-3′ – U: 5′-AAAAAAATTAAGCAGCAGTATCCTCTTGGG-3′ with the L3 primer.
